# Steatitis in Cold-Stunned Kemp’s Ridley Sea Turtles (*Lepidochelys kempii*)

**DOI:** 10.3390/ani11030898

**Published:** 2021-03-21

**Authors:** Rachel C. Turner, Charles J. Innis, Brian A. Stacy, Jorge A. Hernandez, Richard C. Hill, Karen C. Scott, Salvatore Frasca, Michael M. Garner, Rachel E. Burns, Michael D. Arendt, Jennifer Brisson, Terry M. Norton, Sea Rogers Williams, Adam Kennedy, Amy B. Alexander, Nicole I. Stacy

**Affiliations:** 1 Department of Comparative, Diagnostic, and Population Medicine, University of Florida College of Veterinary Medicine, Gainesville, FL 32608, USA; rturner2@ufl.edu (R.C.T.); alexandera@ufl.edu (A.B.A.); 2 New England Aquarium, Boston, MA 02110, USA; cinnis@neaq.org (C.J.I.); akennedy@neaq.org (A.K.); 3 NOAA National Oceanic and Atmospheric Administration, National Marine Fisheries Service, Office of Protected Resources at University of Florida, Gainesville, FL 32611, USA; brian.stacy@noaa.gov; 4 Department of Large Animal Clinical Sciences, University of Florida College of Veterinary Medicine, Gainesville, FL 32608, USA; hernandezja@ufl.edu; 5 Department of Small Animal Clinical Sciences, University of Florida College of Veterinary Medicine, Gainesville, FL 32608, USA; hillr@ufl.edu (R.C.H.); scottkc@ufl.edu (K.C.S.); 6 Connecticut Veterinary Medical Diagnostic Laboratory, Department of Pathobiology and Veterinary Science, University of Connecticut, Storrs, CT 06269, USA; frasca@uconn.edu (S.F.J.); REBurns@sandiegozoo.org (R.E.B.); 7 Northwest ZooPath, Monroe, WA 98272, USA; mikeg@zoopath.com; 8 Marine Resources Division, South Carolina Department of Natural Resources, Charleston, SC 29412, USA; arendtmd@dnr.sc.gov; 9 Massachusetts Veterinary Referral Hospital, Woburn, MA 01801, USA; jbrisson@ethosvet.com; 10 Georgia Sea Turtle Center, Jekyll Island Authority, Jekyll Island, GA 31527, USA; tnorton@jekyllisland.com; 11 National Marine Life Center, Buzzards Bay, MA 02532, USA; rwilliams@nmlc.org

**Keywords:** α-tocopherol, fat necrosis, hypothermia, lipid peroxidation, oxidative stress, pathogenesis, sea turtle, vitamin E

## Abstract

**Simple Summary:**

Kemp’s ridley turtle is the rarest species of sea turtle and is considered to be critically endangered. While the main habitat of adults is the Gulf of Mexico, juveniles forage off the northeastern United States during summer. However, when juveniles fail to leave this area in autumn before water temperatures fall below 10 °C, they become cold-stunned and strand on beaches. Every winter, there are coordinated efforts to rescue and rehabilitate these stranded sea turtles. Cold-stunned sea turtles require supportive care because they suffer from a variety of medical problems, including steatitis, or inflammation of fat tissue. The goal of this study was to further understand steatitis by investigating blood analytes involved with vitamin E metabolism, and investigating microscopic changes that occur in affected fat tissue. We found that various contributing factors may play a role in the development of steatitis. Based on these findings, we conclude that enhanced vitamin E supplementation and dietary modification during rehabilitation may be useful in preventing and treating steatitis. The results from this study will contribute to improving rehabilitation outcomes and successful release of this endangered sea turtle species.

**Abstract:**

The pathogenesis of steatitis that infrequently occurs in cold-stunned Kemp’s ridley sea turtles (KRT; *Lepidochelys kempii*) has been undetermined. The objectives of this study were to investigate the clinical (*n* = 23) and histologic findings (*n* = 11) in cold-stunned KRT, and to compare plasma concentrations of α-tocopherol (vitamin E), thiobarbituric acid reactive substances (TBARS), and the TBARS to vitamin E (T/E) ratio (an assessment of oxidative stress) between cold-stunned KRT with clinically and/or histologically confirmed steatitis (*n* = 10) and free-ranging KRT (*n* = 9). None of the cold-stunned turtles had clinically detectable steatitis at admission, and the median number of days to diagnosis of steatitis was 71 (range 33–469). Histologic findings of affected adipose tissue included heterophilic (*n* = 9) and/or histiocytic (*n* = 5) steatitis, fat necrosis (*n* = 7), myonecrosis (*n* = 2), and intralesional bacteria (*n* = 6). Cold-stunned KRT had significantly lower plasma vitamin E concentrations (median = 3.5 nmol/g), lower plasma TBARS concentrations (median = 1.6 nmol/g), and higher T/E ratios (median = 0.50), than controls (62.3 nmol/g; 2.1 nmol/g; 0.03, respectively). These results suggest a multifactorial etiology for the development of steatitis in KRT during rehabilitation, including tissue injury, septicemia, and various factors resulting in imbalances of anti-/oxidative status. By highlighting the need to provide more effective vitamin E supplementation, and the need to re-assess specific components of the diet, this study may lead to reduced incidence and improved medical management of steatitis in cold-stunned sea turtles.

## 1. Introduction

The Kemp’s ridley sea turtle (KRT; *Lepidochelys kempii*) is a critically endangered species that is primarily found within the Gulf of Mexico and waters of the northwest Atlantic Ocean [1]. The primary population threats are bycatch in various fisheries and poaching of eggs [2]. Their migratory habits and range predispose KRT to cold-stunning because a proportion of immature turtles spend the summer foraging in the waters off the coast of New England, and then migrate south to Cape Hatteras, North Carolina and beyond, in fall and winter [3]. Animals that do not migrate south prior to the onset of winter are at risk of hypothermia from exposure to cold water. As a result, hundreds to thousands of stranded turtles are found on the northeastern and Mid-Atlantic coast of the United States (US) every winter [3,4,5]. Although cold-stunning events are considered natural phenomena with early accounts dating to the 1800s [6], warming sea temperatures may be exacerbating their occurrence in the northeastern US [7].

Depending on the duration and severity of cold exposure, sea turtles are afflicted by acute (e.g., lethargy) and chronic effects (e.g., opportunistic infections) of hypothermia that may require veterinary intervention [8]. In the northeastern US, important sequelae are relatively common and include sepsis, osteomyelitis, ileus, and/or bacterial or fungal pneumonia [8,9,10,11]. In addition, the authors and others have occasionally observed steatitis accompanied by fat necrosis in cold-stunned KRT [12,13], a condition that is relatively uncommon in wild sea turtles that strand due to other causes. However, the cause(s) and lesions of steatitis in cold-stunned turtles have not been thoroughly documented.

Steatitis, in which subcutaneous and intra-abdominal adipose tissues become inflamed, has been reported in several taxa [14,15]. The pathogenesis is not fully understood, but proposed causes include infection, vasculitis, neoplasia, trauma, nutritional deficiencies, toxicosis, environmental factors, and/or immune-mediated disease [16,17]. Steatitis has been documented infrequently in reptiles, including captive American alligators (*Alligator mississippiensis*) with suspected nutritional α-tocopherol (vitamin E) deficiency [18], farmed and wild Nile crocodiles (*Crocodylus niloticus*) with various proposed causes (nutritional vitamin E deficiency, exposure to pollutants, blue-green algae toxicosis, and/or abnormally cold environmental temperatures) [17,19], captive olive ridley sea turtles (*Lepidochelys olivacea*) that were fed frozen-thawed fish without vitamin supplementation [20], and in a wild loggerhead sea turtle (*Caretta caretta*) attributed to possible pollutant-associated toxicosis [15]. It is possible that steatitis and fat necrosis in cold stunned KRT are associated with oxidation caused by vitamin E deficiency as in other species [12,17,19,20]; however, plasma vitamin E concentrations have not been reported and vitamin E is often orally administered during rehabilitation of cold-stunned turtles as part of their dietary supplementation [8].

To describe its occurrence and to investigate the pathogenesis of steatitis in cold-stunned KRT, we summarized the clinical findings, diagnostic imaging, and histologic lesions in a series of cases and evaluated their oxidative state by measuring plasma concentrations of vitamin E and thiobarbituric acid reactive substances (TBARS). As products of lipid peroxidation, TBARS concentrations in blood or tissue could serve as a useful indicator of oxidation in animals with steatitis [21]. In addition, the ratio of TBARS to vitamin E (T/E) may provide a measure of oxidative stress, which occurs when oxidative reactive species exceed the antioxidative capacity of an organism [22]. As these parameters have not been well-studied in sea turtles or other reptiles, we also measured them in free-ranging KRT for comparison to turtles with steatitis.

## 2. Materials and Methods

### 2.1. Ethical Statement

This study was conducted in accordance with Federal Section 10(a)(1)(A) permit 19621, GADNR Scientific Collection Permit (CN21303), FL Marine Turtle Permits MTP-19-163 and MTP 19-021, and UF IACUC# 202006823. Rehabilitation of sea turtles at New England Aquarium is authorized by the United States Fish and Wildlife Service, Permit TE-697823.

### 2.2. Study Animals

Cold-stunned KRT admitted to the New England Aquarium Animal Care Center were included in this study if they were diagnosed with steatitis based on palpation, histologic lesions, and/or computed tomography, and if frozen plasma was available (Table 1). Two turtles were diagnosed with steatitis at secondary facilities to which they had been transferred for completion of rehabilitation, including the Georgia Sea Turtle Center, Jekyll Island, GA, and the National Marine Life Center, Bourne, MA. All turtles received supportive care for cold-stunning per previously described routine protocols [8]. Antibiotics were administered at admission and during rehabilitation as indicated based on the clinical assessment of the attending veterinarian. Antibiotics included ceftazidime (*n* = 18), enrofloxacin (*n* = 13), oxytetracycline (*n* = 12), amikacin (*n* = 10), amoxicillin/clavulanic acid (*n* = 7), ampicillin (*n* = 7), metronidazole (*n* = 3), and/or tobramycin (*n* = 2). During rehabilitation, crab or shrimp were fed occasionally but most of the diet was composed of herring and squid, supplemented daily with Sea Tabs (Pacific Research Labs, Inc. Ramona, CA, USA), at a dose of 50 I.U. vitamin E (d-α-tocopheryl succinate) and 25 mg thiamine per kg of food. At the discretion of the attending veterinarian, three patients also received vitamin E injections during a later stage of treatment when steatitis was diagnosed, based on the suspicion that vitamin E deficiency may have been a contributing factor as seen in other species. These treatments included 1.4 mg vitamin E and 0.07 mg selenium/kg body mass intramuscularly once (one turtle), and once weekly for 3 doses (two turtles) [2.5 mg selenium and 50 mg vitamin E/mL (d-α-tocopheryl acetate), E-Se, Merck Animal Health, Kenilworth, NJ, USA].

The control group consisted of clinically healthy KRT from which plasma samples were obtained when KRT were caught in waters off North Carolina during a health assessment study.

### 2.3. Data Collection

A review of medical records from 2008 to 2018 was performed. Sea turtles were selected based on inclusion criteria for study animals as stated above. Given the retrospective study design, data and sample availability varied among turtles. Records were retrieved from TRACKS^®^ software, and data were organized using Microsoft Excel (Microsoft Corporation, Redmond, WA, USA). The following data were obtained from the hospital record: mass (kg) and straight carapace length (cm) at admission, date of stranding, date of steatitis diagnosis, date of death/euthanasia or release, and, as applicable, physical examination findings, histologic results, and/or necropsy reports. Additional information included blood and tissue culture results, antimicrobial administration, and vitamin supplementations, as available.

### 2.4. Sample Collection

Adipose tissue samples had been previously collected as surgical incisional biopsies or during necropsy examinations. Samples were fixed in formalin, embedded in paraffin, and processed following standard histologic techniques and routine special stains as indicated.

Blood had been collected aseptically from cold-stunned KRT during rehabilitation at admission and during routine examinations using previously described standard protocols [4]. Briefly, blood was collected from the dorsal cervical sinus into heparinized tubes (Microtainer, Becton, Dickinson & Co, Franklin Lakes, NJ, USA). Within 10 min of collection, heparinized whole blood was centrifuged at 1500× *g* for five minutes, and plasma was transferred into cryovials, which were stored at −80 °C for two to nine years.

Blood from control KRT was similarly collected and centrifuged immediately (within <5 min) on the research vessel at 944× *g* for 5 min. Plasma was separated and stored in liquid nitrogen onboard until the end of multi-day overnight research cruises before storage in a shore-based −80 °C freezer for up to three months prior to sample shipment to the University of Florida in 2019. Plasma samples were transferred to a −80 °C freezer upon delivery and remained frozen until analysis.

Whole body computed tomographic imaging (CT) had been completed without sedation using a GE LightSpeed Ultra (GE Healthcare, Chicago, IL, USA), with a slice thickness of 1.3–2.5 mm and constructed in bone and soft tissue algorithms. No contrast agent was used for CT.

### 2.5. Sample Analyses

Vitamin E was quantified as previously described using high-performance liquid chromatography (HPLC) [23], and TBARS were measured with fluorescent spectrophotometry using established methods [24,25,26]. Samples were analyzed in duplicate and results were averaged. Samples that measured below the limit of detection for vitamin E of 7 nmol/g were defined as 3.5 nmol/g (50% of the detection limit) for statistical analysis.

### 2.6. Data Analysis

Plasma concentrations of vitamin E and TBARS, and T/E molar ratio from admission and from available time points during rehabilitation of KRT (*n* = 10) diagnosed with steatitis were compared with data from healthy turtles (*n* = 9). Plasma concentrations of vitamin E, TBARS, and T/E ratio, days to sample collection, and days to diagnosis of steatitis were reported as mean ± SD, or median (minimum, maximum) for non-normally distributed data. The values for mass, SCL, plasma vitamin E, TBARS, and T/E ratio were not normally distributed. The distributions for mass, SCL, and plasma vitamin E, TBARS concentrations, and T/E ratios, were compared between turtles with and without steatitis using Wilcoxon rank sum tests. In all analyses, values of *p* < 0.05 were considered significant.

Trends in vitamin E and TBARS concentrations were visually examined in sea turtles with a diagnosis of steatitis by constructing bar graphs for individual sea turtles sampled three to five times during rehabilitation. A statistical analysis to assess positive or negative linear trends of vitamin E or TBARS concentrations in sea turtles during rehabilitation was not conducted because the number of sea turtles sampled and tested ≥three times for vitamin E or TBARS during rehabilitation was small (9 and 7 sea turtles, respectively) and because the samples collected were not independent (i.e., collected from the same animal).

## 3. Results

Twenty-three KRT with steatitis were reported from 2008–2018, out of a total of 2241 live cold-stunned KRT admitted over that time period, resulting in a prevalence of 1%. Clinical data for each case are provided in Table 1.

**Figure 1:**
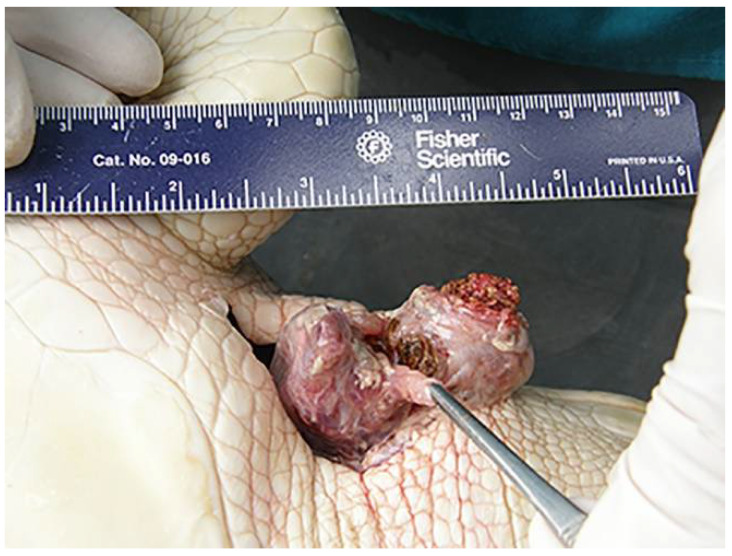
Gross appearance of steatitis in the right cervical region of a Kemp’s ridley sea turtle (*Lepidochelys kempii*) during necropsy. Discolored, necrotic adipose tissue is shown. The turtle is in dorsal recumbency with the head to the right of the image.

**Figure 2:**
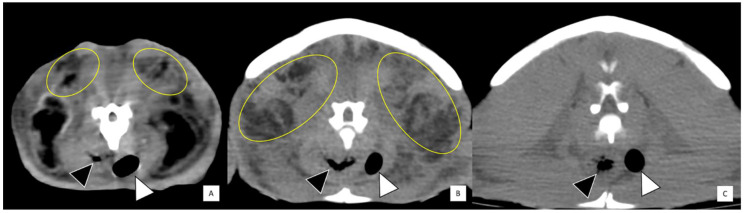
Transverse computed tomographic studies showing heterogeneous soft tissue density (yellow circled areas) consistent with steatitis in the cranial cervical region (**A**) and pre-scapular region (**B**) of two Kemp’s ridley sea turtles (*Lepidochelys kempii*). (**C**) The same turtle shown in (**B**) at approximately the same anatomic location after resolution of disease four months later. Black arrowhead: esophagus, white arrowhead: trachea.

Of 23 cold-stunned KRT, steatitis was diagnosed by one or more of the following methods: palpation (*n* = 18), surgical biopsy initiated after prior palpation (*n* = 4; of which two KRT had two biopsies), postmortem examination (*n* = 5, Figure 1), and/or computed tomography (*n* = 2, Figure 2). Banked plasma was available for 10 of 23 KRT with steatitis. Median number of days to diagnosis of steatitis was 71 (minimum = 33; maximum = 469). Suspected steatitis based on palpation was defined as discrete, firm, nodular subcutaneous swellings, ranging from 1–4 cm in diameter, which were sometimes multifocal (10 of 23), with either two locations (*n* = 7) or three locations (*n* = 3) affected. The cervical region was most commonly affected (*n* = 15), followed by the pre-scapular area (*n* = 9), and/or the inguinal or pre-femoral region (*n* = 8). One turtle had a firm, subcutaneous swelling in the area of the pectoral muscles. CT findings included diffuse heterogeneous mottling of fat within the cervical, pectoral, pre-scapular, and pre-femoral regions, with decreased delineation of normal fascial planes and adjacent muscular margins. In one turtle which had follow-up CT four months after treatment, these findings resolved (Figure 2).

Eighteen of 23 previously cold-stunned KRT were released after successful rehabilitation. Five of 23 turtles either died (*n* = 4) or were euthanized (*n* = 1) due to general clinical decline, and steatitis was considered an incidental finding at the time of death. All three turtles that were treated intramuscularly with vitamin E and selenium were successfully released.

**Figure 3:**
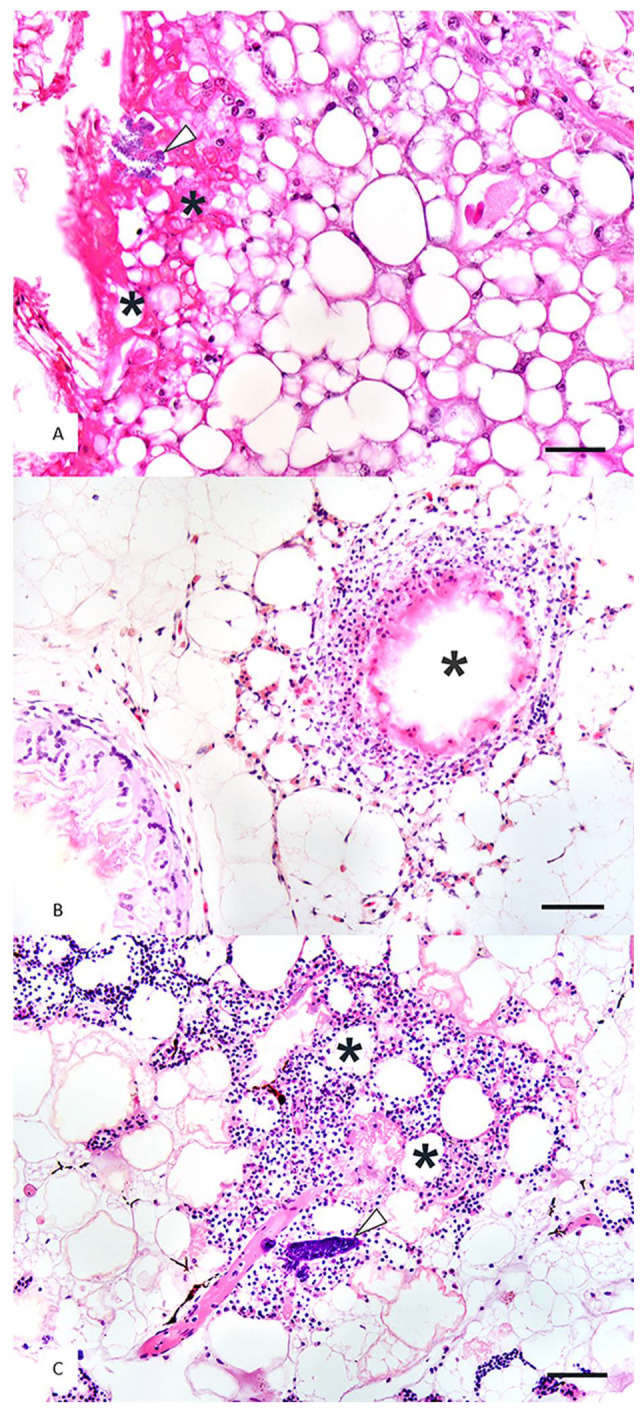
Tissue sections of fat from Kemp’s ridley sea turtles (*Lepidochelys kempii*) with steatitis. Hematoxylin and eosin. (**A**) A cluster of bacteria (arrowhead) is present within an area of necrotic fat and degenerate heterophils (asterisks). Hematoxylin and eosin. ×40 objective. Scale bar = 50 µm. (**B**) Heterophils and macrophages surround necrotic fat (asterisk). A more chronic area of steatitis is present in the lower left as a granuloma composed of multinucleated giant cells formed around ceroid. Hematoxylin and eosin. ×20 objective. Scale bar = 150 µm. (**C**) A bacterial embolus (arrowhead) is within an area of fat necrosis, hemorrhage, and heterophilic inflammation (asterisks). ×20 objective. Scale bar = 170 µm.

The most frequent histologic findings in lesions of adipose tissue in cold-stunned KRT were heterophilic inflammation (9/11), necrosis (7/11), intralesional bacteria (6/11), and histiocytic inflammation (5/11), with all cases having multiple histologic lesions (Table 2, Figure 3). Of the five turtles with necropsy evaluation, cause of death was attributed to pneumonia (*n* = 2); a combination of pneumonia, necrotizing hepatitis, and possible hemolytic anemia (*n* = 1); and presumptive septicemia with evidence of hemolytic anemia and coagulopathy (*n* = 2). One turtle with granulomatous vasculitis in the kidneys and granulomatous hepatitis was diagnosed with mycobacterial pneumonia based on tracheal wash, and one additional turtle was confirmed with *Mycobacterium chelonae* pneumonia via acid fast stain and PCR. Another turtle with fungal hepatitis had subacute to chronic pneumonia with *Pseudomonas*, *Shewenella*, and *Vibrio* isolated from an antemortem tracheal wash. Finally, one turtle had multiple pulmonary granulomas with acid-fast bacilli identified on lung biopsies taken at the same time as biopsies of adipose tissue, but culture and PCR were negative for *Mycobacterium* spp. Notably, one case with septicemia, coagulopathy, and necrosis of adipose tissue also had fat emboli. Additional relevant findings in muscle tissue in five turtles and not listed in Table 2 included myonecrosis (*n* = 3) and heterophilic fasciitis and phlebitis with intralesional bacteria (*n* = 1).

Samples of affected adipose tissue were submitted for culture in four of 23 cases (surgical biopsies *n* = 3; fine needle aspirate *n* = 1). Bacteria were isolated from surgical biopsies of two turtles, including *Serratia marcescens* from one turtle, and multi-drug resistant *Escherichia coli,* and *Enterococcus faecalis* from the other. Sixteen of 23 animals had one to multiple blood cultures submitted adding to a total of 20 cultures of which 10 were positive. Isolated bacteria included *Serratia marcescens* (*n* = 2)*, Enterococcus faecalis* (*n* = 4)*, Citrobacter freundii* (*n* = 2), *Citrobacter braakii* (*n* = 1), and *Escherichia coli* (*n* = 1). In one turtle, *S. marcescens* was the only isolated organism by concurrent culture of abnormal adipose tissue and blood culture. Culture of the adipose tissue was not repeated, but *S. marcescens* was isolated from two subsequent blood cultures.

**Figure 4:**
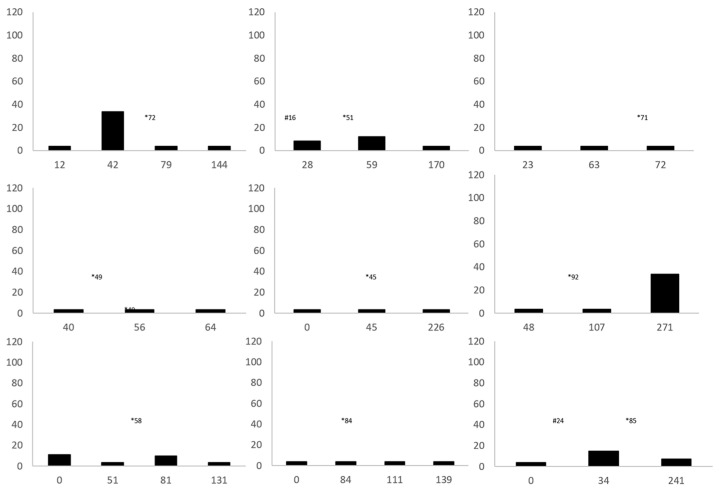
Vitamin E plasma concentrations over time in individual cold-stunned Kemp’s ridley sea turtles (*Lepidochelys kempii*) with steatitis. The *X*-axis is days of rehabilitation, and the *Y*-axis is plasma vitamin E concentrations in nmol/g. * represents the day of diagnosis of steatitis. # represents the day of injectable vitamin E administration for patients that received this treatment (*n* = 2).

**Figure 5:**
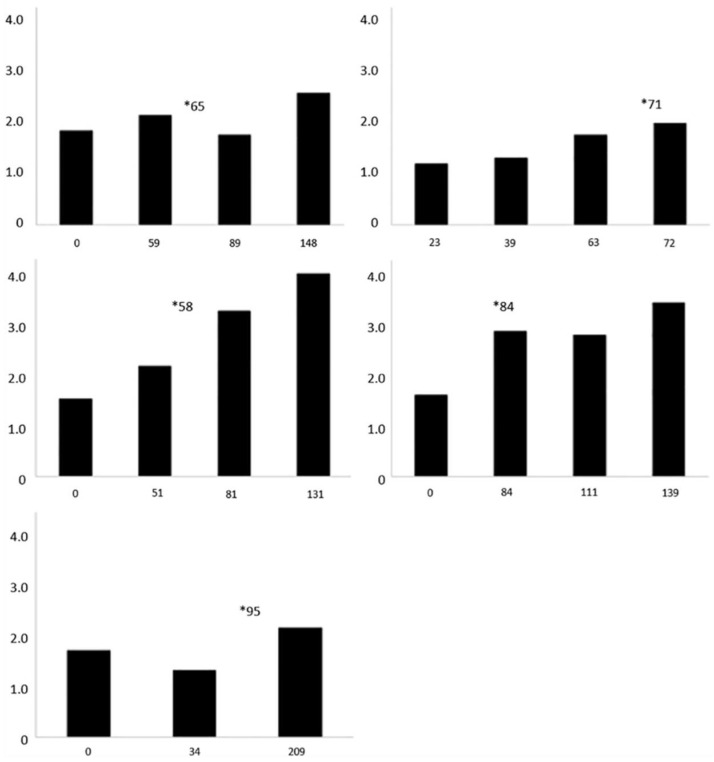
Thiobarbituric acid reactive substances (TBARS) plasma concentrations over time in individual cold-stunned Kemp’s ridley sea turtles (*Lepidochelys kempii*) with steatitis. The *X*-axis is days of rehabilitation, and the *Y*-axis is plasma TBARS concentrations in nmol/g. * represents the day of diagnosis of steatitis.

Mass of cold-stunned KRT was significantly lower and SCL was significantly shorter in cold-stunned KRT, compared to controls; cold-stunned KRT also had lower vitamin E and TBARS concentrations, and higher T/E ratios compared to controls ≤ 0.01 (Table 3). Plasma vitamin E concentrations did not increase linearly during the rehabilitation of eight of nine sea turtles with steatitis that had sufficient volume of plasma banked to measure serial vitamin E concentrations (Figure 4). Plasma TBARS concentration increased linearly during rehabilitation in all seven sea turtles that had enough plasma banked to measure serial TBARS concentrations (Figure 5).

## 4. Discussion

This study reports clinical, histologic, and CT findings in cold-stunned KRT with steatitis, and differences in plasma vitamin E and markers of oxidation between cold-stunned and healthy control KRT. This novel information advances our understanding of the pathophysiology of steatitis in cold-stunned KRT and provides a basis for clinical management decisions during rehabilitation and for future studies.

Based on the circumstances of presentation, clinical, and histologic findings, three potential processes or a combination thereof may contribute to the occurrence of steatitis in cold-stunned KRT. Panniculitis (i.e., steatitis limited to subcutaneous adipose tissue) has been reported in humans exposed to cold temperatures, presumably resulting from vasospasm with consequent impaired blood flow and tissue ischemia [27]. Reperfusion may then lead to lipid peroxidation, oxidative damage, and necrosis [14,28]. Similar mechanisms may cause steatitis in cold-stunned KRT as turtles are gradually warmed at admission and during rehabilitation. Necrotic fat could provide an optimal substrate for colonization by bacteria in turtles with septicemia, a condition that is frequently observed in cold-stunned KRT, thus accounting for concurrent bacterial steatitis [10]. In addition, septicemia secondary to cold-stunning with associated vasculitis and (local or systemic) intravascular coagulation may result in vascular injury or embolic infection that leads to fat necrosis. This is supported by ten positive blood cultures in six turtles and histologic confirmation of bacterial emboli and associated fat necrosis in one turtle. An additional turtle had the same bacteria cultured from adipose and blood (*Serratia marcescens*). Notably, previous or current antimicrobial administration in KRT may have masked the detection of bacteria in histologic sections and/or culture, thus infections may have been underreported. Lastly, vitamin E and TBARS data herein provide evidence that oxidative stress due to cold exposure and/or nutritional deficiency also may contribute to the development of steatitis.

Vitamin E concentrations for control turtles in this study were comparable to other sea turtle species, and provided a baseline for comparison to cold-stunned turtles [29,30,31]. The observed lower plasma vitamin E concentrations in cold-stunned KRT compared to healthy KRT suggest that reduced antioxidative capacity in the face of heightened oxidative stress likely contribute to the onset of steatitis in KRT. Vitamin E is a potent antioxidant that reduces lipid peroxidation in human and animal models [32]. There are several potential explanations for the lower plasma vitamin E concentrations in cold-stunned KRT with steatitis compared to controls. Vitamin E may become diminished during the initial cold-stunning event as tissues respond to diffuse oxidative stress from ischemic damage, similar to reports in humans [33]. Dietary intake of vitamin E may be decreased before cold-stunning events due to reduced food availability or feeding activity in autumn, associated with colder temperatures. Involution of the intestinal mucosa may occur in response to reduced food intake, which would also explain the persistently low vitamin E concentrations despite oral vitamin E supplementation during rehabilitation. In addition, cold-stunned turtles often have gastrointestinal abnormalities, such as ileus, gastritis, and/or enteritis, that may affect absorption of vitamin E [8,9]. Reduced hepatic function is another consideration and is a suggested cause for low blood albumin and BUN concentrations commonly found in cold-stunned KRT at admission [4,34]. Impaired hepatic function may reduce transfer of vitamin E into lipoproteins and thus diminish transport to fatty tissue [35]. Lipoprotein concentrations may also be lower in malnourished animals. Variations in these functional derangements caused by malnutrition may have contributed to the variations in plasma vitamin E concentrations observed during rehabilitation. Returning function with resolution of anorexia could explain in turn, the dramatic increase in vitamin E observed in one turtle which did not receive supplemental vitamin E (Figure 4) [4].

Vitamin E deficiency has been associated with steatitis in various species being fed diets high in poly-unsaturated fatty acids (PUFAs) and deficient in vitamin E, including domestic cats (*Felis catus*) [14,36,37], fish [38,39,40], birds [41,42,43,44], European wild rabbits (*Oryctolagus cuniculus*) [45], marmosets (*Callithrix* spp.) [46], Amazon river dolphins (*Inia geoffrensis*) [47], Nile crocodiles (*Crocodylus niloticus*) [17,19], and olive ridley sea turtles (*Lepidochelys olivacea*) [20]. The proposed mechanism of vitamin E deficiency-associated steatitis is that free radicals are produced when PUFAs are metabolized in the absence of sufficient vitamin E to quench the production of free radicals and, as a result, proteins, DNA, and fat become oxidized. The captive diet of KRT while in rehabilitation may contribute to low vitamin E concentrations and oxidative stress, either by direct deficiency of dietary vitamin E, or inappropriate dietary levels of other nutrients that may affect vitamin E metabolism, such as vitamin A. Free-ranging KRT primarily consume mollusks and crustaceans in their natural diet, which contain relatively low concentrations of polyunsaturated and other fats, so the amount of vitamin E needed to prevent oxidation may be low in free-ranging turtles [3,48]. During rehabilitation, however, KRT are commonly fed herring, containing 3–12 g crude fat (on a fed basis), and 2.1 g PUFA/100 g, whereas crab contain 0.9–1 g crude fat, 0.4 g PUFA/100 g and clams contain 0.6 g crude fat, 0.2 g PUFA/100 g) [48,49,50,51]. Feeding herring increases a turtle’s requirement for vitamin E to prevent oxidation of the polyunsaturated bonds in PUFA.

Cold-stunned KRT had significantly higher T/E ratios compared to controls because vitamin E concentrations were lower than controls, not because TBARS concentrations were high. On the contrary, TBARS concentrations in cold stressed KRT were significantly lower than in controls, increased only a small amount during rehabilitation and never increased above plasma TBARS concentrations of healthy turtles. These findings suggest poor tissue perfusion at admission and/or that lipid peroxidation may have developed during rehabilitation and was not present at admission. The increase in TBARS concentrations over time may reflect reperfusion injury secondary to improved tissue perfusion after admission. It is possible also that vitamin E supplementation was sufficient to reduce TBARS concentrations but total plasma concentrations of vitamin E did not increase because vitamin E was consumed in the process. It is also possible that TBARS in plasma is a poor marker of lipid peroxidation in reptiles. The observed lower plasma concentrations of vitamin E and TBARS in cold-stressed KRT compared to controls may have resulted from differences in environment and activity. Healthy turtles were larger in mass and size, sampled at sea during summer months when they were metabolically active, feeding on their natural diet, and thus were in optimal metabolic condition, whereas cold-stressed turtles were sampled in captivity in autumn and winter when turtles may have been less metabolically active and feeding on a captive diet.

The diagnosis of steatitis was made, on average, when turtles were two months into rehabilitation. It is possible, therefore, that the effects from reperfusion injury and/or oxidative damage may take time to manifest after admission, depending on the severity and extent of insult to adipose tissue. It was notable that two turtles were diagnosed with steatitis at least 14 months into rehabilitation. This may suggest a slowly developing, subclinical nutritional component in addition to other predisposing factors. Other potential contributing factors include inflammation secondary to venipuncture of the vasculature of the neck, administration of subcutaneous fluids, or injection of antibiotics and other medications [52]. These factors, though, do not adequately explain the occurrence of steatitis in regions that had not been used for injections, regions distant from injection sites (e.g., retrocoelomic fat), nor the lack of steatitis reports from facilities in warmer geographic locations that also give numerous injections to sea turtles.

Steatitis is often found concurrently with additional pathologic findings in other species, including myopathy, encephalomalacia, and hepatic necrosis [39,42,46]. In dogs and humans, panniculitis can occur with concurrent pancreatitis or pancreatic neoplasia; leakage of pancreatic enzymes into the systemic circulation has been suspected to be associated with these conditions but this remains unconfirmed [53,54,55]. Pancreatic atrophy resulting in fat malabsorption has been suggested as the cause of vitamin E deficiency and resulting steatitis in non-human primates [46]. In contrast to these observations in mammals, pancreatitis was absent in all five KRT which were examined post-mortem in this report. In addition to systemic pathologic findings, steatitis lesions are thought to potentially be painful, since cats [14], fish [40] and crocodiles [19] with steatitis have been reportedly lethargic, anorexic, and reluctant to move or swim. Thus, steatitis may contribute to clinical debilitation in affected turtles.

Many of the cases reported here were not confirmed by histopathology. Histopathology was pursued most often earlier in the timespan covered by this study. Upon acquiring clinical experience with histologically confirmed cases, clinicians often made the presumptive diagnosis of steatitis based on clinical presentation, avoiding biopsy. It cannot be determined whether any of these presumptive cases were truly steatitis versus other types of lesions, such as granulomas or injection site reactions. Nonetheless, based on the similarity of presentation of the presumptive cases and the confirmed cases, the presumptive diagnosis was likely to be accurate.

The limited availability and duration of storage of previously collected and banked plasma samples in KRT with steatitis are important limitations of this study. Whereas medical record data were available for 23 turtles, there was only sufficient banked plasma to measure paired vitamin E and TBARS in 10 turtles. In addition, there was a difference in storage time between the samples in KRT with steatitis (2–10 years) and control turtles (5–6 months). However, vitamin E reportedly remains stable in plasma for up to 15 years when stored at −70 °C or colder, and the oldest sample in this study was stored for up to 10 years [56,57]. Thiobarbituric acid reactive substances have been shown to be stable for at least 30 days when samples are kept frozen [58] and have been stable for at least three years when samples were kept at −80 °C (personal observation KCS). It is possible that lower plasma TBARS in steatitis turtles resulted from some degree of degradation during storage, but vitamin E concentrations should not have been affected. There were no overt trends of vitamin E or TBARS concentrations associated with length of sample storage. Future studies using fresh plasma samples from KRT with steatitis may be useful to further understand TBARS dynamics in turtles affected by this condition.

It is important to note that this study did not evaluate vitamin E and TBARS concentrations in cold-stunned turtles that were not diagnosed with steatitis. It is possible that low vitamin E and TBARS concentrations occur in cold-stunned turtles, in general, not only those affected by steatitis. However, the low prevalence of 1% in cold-stunned KRT suggests that multiple factors contribute to the development of steatitis. Evaluation of vitamin E and TBARS concentrations in a larger cohort of cold-stunned turtles would be worthwhile, including turtles with no evidence of steatitis. Measurement of adipose tissue concentrations of vitamin E and TBARS may also provide additional information.

## 5. Conclusions

The findings of this study suggest that steatitis in cold-stunned KRT may be a multifactorial disease process that includes tissue injury, septicemia, and imbalances of anti-oxidative status. Plasma vitamin E concentrations were consistently low despite supplementation; thus, there is need to develop more effective vitamin E supplementation protocols for cold-stunned turtles (i.e., evaluation for effective dose, formulation [e.g., products with or without selenium], route, and frequency) and to further investigate other potentially contributing factors (e.g., immunosuppression, altered microbiome). Dietary adjustment to reduce PUFA intake may also be warranted. Further collaboration between clinicians and veterinary nutritionists is warranted to improve captive sea turtle diets.

## Figures and Tables

**Table 1:** Overview of cold-stunned Kemp’s ridley sea turtles (*Lepidochelys kempii*) admitted for rehabilitation with diagnosis of steatitis included in this study. NP = not performed; NEG = negative; POS = positive; B = biopsy; PM = post-mortem tissue sample.

ID	Steatitis Diagnosis by Palpation (Y/N)	Histopathology	Day of Steatitis Diagnosis after Admission	Blood Culture	Tissue Culture	Plasma Vitamin E (Y/N)	Plasma TBARS (Y/N)	Paired Vitamin E and TBARS Samples (Y/N)	Outcome
1	Y	NP	72	NEG	NP	Y	N	N	Released
2	N	PM	33	NP	NP	Y	N	N	Died
3 ^2^	Y	NP	51	NEG	NP	Y	N	N	Released
4	Y	NP	50	POS	POS	N	N	N	Released
5	N	PM	469	NP	NP	Y	Y	Y	Euthanized
6	N	PM	84	NP	NP	N	Y	N	Died
7 ^1^	Y	NP	71	NEG	NP	Y	Y	Y	Released
8	Y	NP	65	NEG	NP	Y	Y	Y	Released
9	Y	NP	70	POS	NP	N	N	N	Released
10	Y	B (*n* = 2)	55	NEG	POS	Y	Y	Y	Released
11 ^1^	Y	B	49	NEG	NP	Y	Y	N	Released
12	Y	NP	85	NEG	NP	Y	Y	Y	Released
13	Y	NP	36	NP	NEG	Y	N	N	Released
14	Y	NP	45	NP	NP	Y	N	N	Released
15	Y	NP	92	POS	NP	Y	Y	Y	Released
16	Y	NP	84	NEG	NP	Y	Y	Y	Released
17	Y	NP	58	NP	NP	Y	Y	Y	Released
18 ^2^	Y	B (*n* = 2)	115	POS (*n* = 2)	NEG	Y	N	N	Released
19	N	PM	437	NP	NP	N	N	N	Died
20 ^2^	Y	B	75	NEG	NP	Y	N	N	Released
21	Y	NP	95	POS (*n* = 3)	NP	Y	Y	Y	Released
22	Y	NP	45	NEG	NP	Y	Y	Y	Released
23	N	PM	89	POS (*n* = 2)	NP	N	N	N	Died

^1^ Diagnosis of steatitis via computed tomography. ^2^ Vitamin E supplementation (injectable).

**Table 2:** Histopathologic findings in steatitis lesions of nine cold-stunned Kemp’s ridley sea turtles (*Lepidochelys kempii*) examined by surgical biopsy (*n* = 6) or post-mortem (*n* = 5).

Histopathology ID #	Patient #	Necropsy or Biopsy	Heterophilic Inflammation	Necrosis of Adipose Tissue	Bacteria Present	Histiocytic Inflammation	Myo- Necrosis	Thrombi	Minerali-Zation
1	1	Biopsy	Y	Y	Y	Y	N	N	N
2	1	Biopsy	Y	Y	Y	Y	N	N	N
3	2	Biopsy	N	Y	N	N	Y	N	Y
4	3	Biopsy	Y	Y	N	N	N	N	N
5	3	Biopsy	N	Y	Y	N	N	N	N
6	4	Biopsy	Y	N	N	Y	N	N	N
7	5	Necropsy	Y	N	Y	N	Y	Y	N
8	6	Necropsy	Y	N	N	Y	N	N	N
9	7	Necropsy	Y	N	Y	Y	N	N	N
10	8	Necropsy	Y	Y	Y	N	N	N	N
11	9	Necropsy	Y	Y	N	N	N	N	N
Total			9	7	6	5	2	1	1

**Table 3:** Mass, straight carapace length, and plasma concentrations of α-tocopherol (vitamin E), thiobarbituric acid reactive substances (TBARS), and TBARS to vitamin E ratios of cold-stunned Kemp’s ridley sea turtles (*Lepidochelys kempii*) affected with steatitis compared to those of free-ranging immature control turtles.

Variables	Cold-Stunned Turtles with Diagnosis of Steatitis	Free-Ranging Immature Control Turtles	Z	*p*
**Mass (kg)**				
N	10	8		
Mean ± SD	2.9 ± 0.9	17.9 ± 7.8		
Median (minimum, maximum)	3.1 (1.6, 4.1)	18.1 (3.4, 28.0)	3.20	<0.01
**Straight carapace length (cm)**				
N	10	8		
Mean ± SD	27.5 ± 3.1	46.1 ± 8.7		
Median (minimum, maximum)	28.5 (22.6, 31.4)	47.7 (27.0, 55.3)	2.98	<0.01
Vitamin E * nmol/g				
N	10	9		
Mean ± SD	3.6 ± 0.7	61.2 ± 23.3		
Median (minimum, maximum)	3.5 (2.3, 5.5)	62.3 (25.3, 90.9)	3.67	<0.01
**TBARS nmol/g**				
N	10	9		
Mean ± SD	1.8 ± 0.6	2.5 ± 0.7		
Median (minimum, maximum)	1.6 (1.2, 3.2)	2.1 (1.8, 3.9)	2.41	0.01
**TBARS to vitamin E ratio**				
N	10	9		
Mean ± SD	0.52 ± 0.18	0.05 ± 0.03		
Median (minimum, maximum)	0.50 (0.28, 0.92)	0.03 (0.02, 0.10)	3.63	<0.01

* The lowest measurable quantity of vitamin E was 7.0 nmol/g. Samples that measured below the level of detection were defined as 3.5 nmol/g.

## Data Availability

The data presented in this study are available on request from the corresponding author.

## References

[1] Wibbels T., Bevan E. Lepidochelys kempii (Errata Version Published in 2019).

[2] National Marine Fisheries Service, U.S. Fish and Wildlife Service, SEMARNAT Fish and Wildlife Service; SEMARNAT. Bi-National Recovery Plan for the Kemp’s Ridley Sea Turtle (Lepidochelys kempii).

[3] Witherington B.E., Manire C.A., Norton T.M., Stacy B.A., Innis C.J., Harms C.A. (2017). Sea Turtles in Context: Their Life History and Conservation. Sea Turtle Health and Rehabilitation.

[4] Innis C.J., Ravich J.B., Tlusty M.F., Hoge M.S., Wunn D.S., Boerner-Neville L.B., Merigo C., Weber E.S. (2009). Hematologic and Plasma Biochemical Findings in Cold-Stunned Kemp’s Ridley Turtles: 176 cases (2001–2005). J. Am. Vet. Med. Assoc..

[5] Hunt K.E., Innis C., Rolland R.M. (2012). Corticosterone and Thyroxine in Cold-Stunned Kemp’s Ridley Sea Turtles (*Lepidochelys kempii*). J. Zoo Wildl. Med..

[6] Witherington B.E., Ehrhart L.M. (1989). Hypothermic Stunning and Mortality of Marine Turtles in the Indian River Lagoon System, Florida. Copeia.

[7] Griffin L.P., Griffin C.R., Finn J.T., Prescott R.L., Faherty M., Still B.M., Danylchuk A.J. (2019). Warming Seas Increase Cold-Stunning Events for Kemp’s Ridley Sea Turtles in the Northwest Atlantic. PLoS ONE.

[8] Innis C.J., Staggs L.A., Manire C.A., Norton T.M., Stacy B.A., Innis C.J., Harms C.A. (2017). Cold-Stunning. Sea Turtle Health and Rehabilitation.

[9] Innis C., Nyaoke A.C., Williams C.R., Dunnigan B., Merigo C., Woodward D.L., Weber E.S., Frasca S. (2009). Pathologic and Parasitologic Findings of Cold-Stunned Kemp’s Ridley Sea Turtles (*Lepidochelys kempii*) Stranded on Cape Cod, Massachusetts, 2001–2006. J. Wildl. Dis..

[10] Innis C.J., Braverman H., Cavin J.M., Ceresia M.L., Baden L.R., Kuhn D.M., Frasca S., McGowan J.P., Hirokawa K., Weber E.S. (2014). Diagnosis and Management of Enterococcus spp Infections During Rehabilitation of Cold-Stunned Kemp’s Ridley Turtles (*Lepidochelys kempii*): 50 cases (2006–2012). J. Am. Vet. Med. Assoc..

[11] Powell A.L., Tuxbury K.A., Cavin J.M., Stacy B.A., Frasca S., Stacy N.I., O’Sullivan Brisson J., Williams S.R., Mccarthy R.J., Innis C.J. (2021). Osteomyelitis in Rehabilitating Cold-Stunned Kemp’s Ridley Sea Turtles (*Lepidochelys kempii*): 25 cases (2008–2018). J. Am. Vet. Med. Assoc..

[12] Mumford S.L., Johnson L., Herbst L. Multifocal Steatitis Lesions in a Kemp’s Ridley Sea Turtle (*Lepidochelys kempii*). Proceedings of the 1999 IAAAM Conference.

[13] Innis C.J., Ceresia M.L., Merigo C., Scott Weber E., Papich M.G. (2012). Single-Dose Pharmacokinetics of Ceftazidime and Fluconazole During Concurrent Clinical Use in Cold-Stunned Kemp’s Ridley Turtles (*Lepidochelys kempii*). J. Vet. Pharmacol. Ther..

[14] Miller W.H., Griffin C.E., Campbell K.L. (2013). Nutrition and Skin Disease. Muller and Kirk’s Small Animal Dermatology.

[15] Oros J., Monagas P., Calabuig P., Luzardo O.P., Camacho M. (2013). Pansteatitis Associated with High Levels of Polychlorinated Biphenyls in a Wild Loggerhead Sea Turtle *Caretta caretta*. Dis. Aquat. Org..

[16] German A.J., Foster A.P., Holden D., Hotston Moore A., Day M.J., Hall E.J. (2003). Sterile Nodular Panniculitis and Pansteatitis in Three Weimaraners. J. Small Anim. Pract..

[17] Lane E.P., Huchzermeyer F.W., Govender D., Bengis R.G., Buss P.E., Hofmeyr M., Myburgh J.G., Steyl J.C., Pienaar D.J., Kotze A. (2013). Pansteatitis of Unknown Etiology Associated with Large-Scale Nile Crocodile (*Crocodylus niloticus*) Mortality in Kruger National Park, South Africa: Pathologic Findings. J. Zoo Wildl. Med..

[18] Larsen R.E., Buergelt C., Cardeilhac P.T., Jacobson E.R. (1983). Steatitis and Fat Necrosis in Captive Alligators. J. Am. Vet. Med. Assoc..

[19] Osthoff G., Hugo A., Bouwman H., Buss P., Govender D., Joubert C.C., Swarts J.C. (2010). Comparison of the Lipid Properties of Captive, Healthy Wild, and Pansteatitis-Affected Wild Nile Crocodiles (*Crocodylus niloticus*). Comp. Biochem. Physiol. Part A Mol. Integr. Physiol..

[20] Manawatthana S., Kasorndorkbua C. Steatitis and Vitamin E Deficiency in Captive Olive Ridley Turtles (*Lepidochelys olivacea*). Proceedings of the 2nd International Symposium on SEASTAR2000 and Asian Bio-logging Science.

[21] Valdivia P.A., Zenteno-Savin T., Gardner S.C., Aguirre A.A. (2007). Basic Oxidative Stress Metabolites in Eastern Pacific Green Turtles (*Chelonia mydas agassizii*). Comp. Biochem. Physiol. Part C Toxicol. Pharmacol..

[22] Pizzino G., Irrera N., Cucinotta M., Pallio G., Mannino F., Arcoraci V., Squadrito F., Altavilla D., Bitto A. (2017). Oxidative Stress: Harms and Benefits for Human Health. Oxid. Med. Cell. Longev..

[23] Browne R.W., Armstrong D., Armstrong D. (1998). Simulatneous Determination of Serum Retinol, Tocopherols, and Carotenoids by HPLC. Methods in Molecular Biology: Free Radical and Antioxidant Protocols.

[24] Armstrong D., Hiramitsu T., Ueda T. (1998). In Vitro Screening for Antioxidant Activity. Methods Mol. Biol..

[25] Miller N., Armstrong D. (1998). Nonvitamin Plasma Antioxidants. Methods in Molecular Biology: Free Radical and Antioxidant Protocols.

[26] Yagi K. (1998). Simple Assay for the Level of Total Lipid Peroxides in Serum or Plasma. Methods Mol. Biol..

[27] Aroni K., Aivaliotis M., Tsele E., Charalambopoulos D., Davaris P. (1998). An Unusual Panniculitis Appearing in the Winter with Good Response to Tetracycline. J. Dermatol..

[28] Milei J., Forcada P., Fraga C.G., Grana D.R., Iannelli G., Chiariello M., Tritto I., Ambrosio G. (2007). Relationship Between Oxidative Stress, Lipid Peroxidation, and Ultrastructural Damage in Patients with Coronary Artery Disease Undergoing Cardioplegic Arrest/Reperfusion. Cardiovasc. Res..

[29] Frutchey K. (2004). Plasma Levels of Vitamin A and E in Marine Turtles (*Chelonia mydas* and *Caretta caretta*). Master’s Thesis.

[30] Deem S.L., Dierenfeld E.S., Sounguet G.P., Alleman A.R., Cray C., Poppenga R.H., Norton T.M., Karesh W.B. (2006). Blood Values in Free-Ranging Nesting Leatherback Sea Turtles (*Dermochelys coriacea*) on the Coast of the Republic of Gabon. J. Zoo Wildl. Med..

[31] Innis C., Merigo C., Dodge K., Tlusty M., Dodge M., Sharp B., Myers A., McIntosh A., Wunn D., Perkins C. (2010). Health Evaluation of Leatherback Turtles (*Dermochelys coriacea*) in the Northwestern Atlantic During Direct Capture and Fisheries Gear Disentanglement. Chelonian Conserv. Biol..

[32] Singh U., Devaraj S., Jialal I. (2005). Vitamin E, Oxidative Stress, and Inflammation. Annu. Rev. Nutr..

[33] Suantawee T., Tantavisut S., Adisakwattana S., Tanavalee A., Yuktanandana P., Anomasiri W., Deepaisarnsakul B., Honsawek S. (2013). Oxidative Stress, Vitamin E, and Antioxidant Capacity in Knee Osteoarthritis. J. Clin. Diagn. Res..

[34] Drevon C.A. (1991). Absorption, Transport and Metabolism of Vitamin E. Free Radic. Res. Commun..

[35] Chung S., Ghelfi M., Atkinson J., Parker R., Qian J., Carlin C., Manor D. (2016). Vitamin E and Phosphoinositides Regulate the Intracellular Localization of the Hepatic α-Tocopherol Transfer Protein. J. Biol. Chem..

[36] Niza M., Vilela C., Ferreira L. (2003). Feline Pansteatitis Revisited: Hazards of Unbalanced Home-Made Diets. J. Feline Med. Surg..

[37] Fytianou A., Koutinas A.F., Saridomichelakis M.N., Koutinas C.K. (2006). Blood Alpha-Tocopherol, Selenium, and Glutathione Peroxidase Changes and Adipose Tissue Fatty Acid Changes in Kittens with Experimental Steatitis (Yellow Fat Disease): A Comparative Study Between the Domestic Shorthaired and Siamese Breed. Biol. Trace Elem. Res..

[38] Bricknell I.R., Bruno D.W., Bowden T.J., Smith P. (1996). Fat Cell Necrosis Syndrome in Atlantic Halibut, *Hippoglossus hippoglossus* L.. Aquaculture.

[39] Guarda F., Bertoja G., Zoccarato I., Tartari E., Biolatti B. (1997). Spontaneous Steatitis of Epicardial Fat in Farmed White Sturgeon (*Acipenser transmontanus*). Aquaculture.

[40] Roberts R.J., Agius C. (2008). Pan-Steatitis in Farmed Northern Bluefin Tuna, *Thunnus thynnus* (L.), in the Eastern Adriatic. J. Fish Dis..

[41] Dierenfeld E.S. (1989). Vitamin E Deficiency in Zoo Reptiles, Birds, and Ungulates. J. Zoo Wildl. Med..

[42] Pollock C.G., Sleeman J.M., Houle C.D., Ramsay E.C. (1999). Vitamin E Deficiency and Pansteatitis in Juvenile Boat-Billed Herons (*Cochlearius cochlearius*). J. Zoo Wildl. Med..

[43] Wong E., Mikaelian I., Desnoyers M., Fitzgerald G. (1999). Pansteatitis in a Free-Ranging Red-Tailed Hawk (*Buteo jamaicensis*). J. Zoo Wildl. Med..

[44] Myburgh J., Botha A. (2009). Decline in Herons Along the Lower Olifants River—Could Pansteatitis Be a Contributing Factor?. Vet News.

[45] Jones D., Howard A.N., Gresham G.A. (1969). Aetiology of “Yellow Fat” Disease (Pansteatitis) in the Wild Rabbit. J. Comp. Pathol..

[46] Juan-Salles C., Prats N., Resendes A., Domingo M., Hilton D., Ruiz J.M., Garner M.M., Valls X., Marco A.J. (2003). Anemia, Myopathy, and Pansteatitis in Vitamin E-Deficient Captive Marmosets (Callithrix spp.). Vet. Pathol..

[47] Bonar C.J., Wagner R.A. (2003). A Third Report of “Golf Ball Disease” in an Amazon River Dolphin (*Inia geoffrensis*) Associated with Streptococcus iniae. J. Zoo Wildl. Med..

[48] Hoopes L.A., Koutsos E.A., Manire C.A., Norton T.M., Stacy B.A., Innis C.J., Harms C.A. (2017). Nutrition. Sea Turtle Health and Rehabilitation.

[49] Herring, Raw. https://ndb.nal.usda.gov//fdc-app.html#/food-details/782531/nutrients.

[50] Clams, Raw. https://ndb.nal.usda.gov//fdc-app.html#/food-details/782757/nutrients.

[51] Crustaceans, Crab, Blue, Raw. https://ndb.nal.usda.gov//fdc-app.html#/food-details/174204/nutrients.

[52] Innis C., Kennedy A., Wocial J., Burgess E., Papich M.G. (2020). Comparison of Oxytetracycline Pharmacokinetics After Multiple Subcutaneous Injections in Three Sea Turtle Species. J. Herpetol. Med. Surg..

[53] Mellanby R.J., Stell A., Baines E., Chantrey J.C., Herrtage M.E. (2003). Panniculitis Associated with Pancreatitis in a Cocker Spaniel. J. Small Anim. Pract..

[54] Mourad F.H., Hannoush H.M., Bahlawan M., Uthman I., Uthman S. (2001). Panniculitis and Arthritis as the Presenting Manifestation of Chronic Pancreatitis. J. Clin. Gastroenterol..

[55] O’Kell A.L., Inteeworn N., Diaz S.F., Saunders G.K., Panciera D.L. (2010). Canine Sterile Nodular Panniculitis: A Retrospective Study of 14 Cases. J. Vet. Intern. Med..

[56] Comstock G.W., Alberg A.J., Helzlsouer K.J. (1993). Reported Effects of Long-Term Freezer Storage on Concentrations of Retinol, Beta-Carotene, and Alpha-Tocopherol in Serum or Plasma Summarized. Clin. Chem..

[57] Brown Thomas J., Duewer D.L., Kline M.C., Sharpless K.E. (1998). The Stability of Retinol, Α-Tocopherol, Trans-Lycopene, and Trans-Β-Carotene in Liquid-Frozen and Lyophilized Serum. Clin. Chim. Acta.

[58] Young I.S., Trimble E.R. (1991). Measurement of Malondialdehyde in Plasma by High Performance Liquid Chromatography with Fluorimetric Detection. Ann. Clin. Biochem..

