# Correction: Turner et al. Steatitis in Cold-Stunned Kemp’s Ridley Sea Turtles (*Lepidochelys kempii*). *Animals* 2021, *11*, 898

**DOI:** 10.3390/ani12091129

**Published:** 2022-04-28

**Authors:** Rachel C. Turner, Charles J. Innis, Brian A. Stacy, Jorge A. Hernandez, Richard C. Hill, Karen C. Scott, Salvatore Frasca, Michael M. Garner, Rachel E. Burns, Michael D. Arendt, Jennifer Brisson, Terry M. Norton, Sea Rogers Williams, Adam Kennedy, Amy B. Alexander, Nicole I. Stacy

**Affiliations:** 1 Department of Comparative, Diagnostic, and Population Medicine, University of Florida College of Veterinary Medicine, Gainesville, FL 32608, USA; rturner2@ufl.edu (R.C.T.); alexandera@ufl.edu (A.B.A.); 2 New England Aquarium, Boston, MA 02110, USA; cinnis@neaq.org (C.J.I.); akennedy@neaq.org (A.K.); 3 NOAA National Oceanic and Atmospheric Administration, National Marine Fisheries Service, Office of Protected Resources at University of Florida, Gainesville, FL 32611, USA; brian.stacy@noaa.gov; 4 Department of Large Animal Clinical Sciences, University of Florida College of Veterinary Medicine, Gainesville, FL 32608, USA; hernandezja@ufl.edu; 5 Department of Small Animal Clinical Sciences, University of Florida College of Veterinary Medicine, Gainesville, FL 32608, USA; hillr@ufl.edu (R.C.H.); scottkc@ufl.edu (K.C.S.); 6 Connecticut Veterinary Medical Diagnostic Laboratory, Department of Pathobiology and Veterinary Science, University of Connecticut, Storrs, CT 06269, USA; frasca@uconn.edu (S.F.J.); reburns@sandiegozoo.org (R.E.B.); 7 Northwest ZooPath, Monroe, WA 98272, USA; mikeg@zoopath.com; 8 Marine Resources Division, South Carolina Department of Natural Resources, Charleston, SC 29412, USA; arendtmd@dnr.sc.gov; 9 Massachusetts Veterinary Referral Hospital, Woburn, MA 01801, USA; jbrisson@ethosvet.com; 10 Georgia Sea Turtle Center, Jekyll Island Authority, Jekyll Island, GA 31527, USA; tnorton@jekyllisland.com; 11 National Marine Life Center, Buzzards Bay, MA 02532, USA; rwilliams@nmlc.org

## Error in Figure/Table

In the original publication [1], there was a mistake in Table 3 and Figure 4 as published. After publication, the authors found an error in the spreadsheet used to calculate vitamin E concentrations. This resulted in all vitamin E concentrations being several-fold lower than they should have been. Corrected concentrations and ratios are provided in the new table. Because all previously reported concentrations were calculated using the same error, relative concentrations and the conclusions on which they are based have not changed.

The corrected Table 3 and Figure 4 appear below. The Abstract was updated with corrected data.

**Abstract:** The pathogenesis of steatitis that infrequently occurs in cold-stunned Kemp’s ridley sea turtles (KRT; *Lepidochelys kempii*) has been undetermined. The objectives of this study were to investigate the clinical (*n* = 23) and histologic findings (*n* = 11) in cold-stunned KRT, and to compare plasma concentrations of α-tocopherol (vitamin E), thiobarbituric acid reactive substances (TBARS), and the TBARS to vitamin E (T/E) ratio (an assessment of oxidative stress) between cold-stunned KRT with clinically and/or histologically confirmed steatitis (*n* = 10) and free-ranging KRT (*n* = 9). None of the cold-stunned turtles had clinically detectable steatitis at admission, and the median number of days to diagnosis of steatitis was 71 (range 33–469). Histologic findings of affected adipose tissue included heterophilic (*n* = 9) and/or histiocytic (*n* = 5) steatitis, fat necrosis (*n* = 7), myonecrosis (*n* = 2), and intralesional bacteria (*n* = 6). Cold-stunned KRT had significantly lower plasma vitamin E concentrations (median = 3.5 nmol/g), lower plasma TBARS concentrations (median = 1.6 nmol/g), and higher T/E ratios (median = 0.50), than controls (62.3 nmol/g; 2.1 nmol/g; 0.03, respectively). These results suggest a multifactorial etiology for the development of steatitis in KRT during rehabilitation, including tissue injury, septicemia, and various factors resulting in imbalances of anti-/oxidative status. By highlighting the need to provide more effective vitamin E supplementation, and the need to re-assess specific components of the diet, this study may lead to reduced incidence and improved medical management of steatitis in cold-stunned sea turtles.

**Figure 4:**
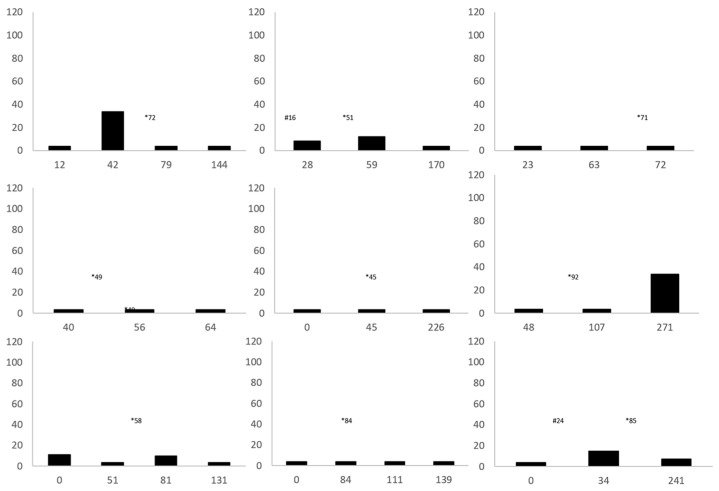
Vitamin E plasma concentrations over time in individual cold-stunned Kemp’s ridley sea turtles (*Lepidochelys kempii*) with steatitis. The *X*-axis is days of rehabilitation, and the *Y*-axis is plasma vitamin E concentrations in nmol/g. * represents the day of diagnosis of steatitis. # represents the day of injectable vitamin E administration for patients that received this treatment (*n* = 2).

**Table 3:** Mass, straight carapace length, and plasma concentrations of α-tocopherol (vitamin E), thiobarbituric acid reactive substances (TBARS), and TBARS to vitamin E ratios of cold-stunned Kemp’s ridley sea turtles (*Lepidochelys kempii*) affected with steatitis compared to those of free-ranging immature control turtles.

Variables	Cold-Stunned Turtles with Diagnosis of Steatitis	Free-Ranging Immature Control Turtles	Z	*p*
**Mass (kg)**				
N	10	8		
Mean ± SD	2.9 ± 0.9	17.9 ± 7.8		
Median (minimum, maximum)	3.1 (1.6, 4.1)	18.1 (3.4, 28.0)	3.20	<0.01
**Straight carapace length (cm)**				
N	10	8		
Mean ± SD	27.5 ± 3.1	46.1 ± 8.7		
Median (minimum, maximum)	28.5 (22.6, 31.4)	47.7 (27.0, 55.3)	2.98	<0.01
Vitamin E * nmol/g				
N	10	9		
Mean ± SD	3.6 ± 0.7	61.2 ± 23.3		
Median (minimum, maximum)	3.5 (2.3, 5.5)	62.3 (25.3, 90.9)	3.67	<0.01
**TBARS nmol/g**				
N	10	9		
Mean ± SD	1.8 ± 0.6	2.5 ± 0.7		
Median (minimum, maximum)	1.6 (1.2, 3.2)	2.1 (1.8, 3.9)	2.41	0.01
**TBARS to vitamin E ratio**				
N	10	9		
Mean ± SD	0.52 ± 0.18	0.05 ± 0.03		
Median (minimum, maximum)	0.50 (0.28, 0.92)	0.03 (0.02, 0.10)	3.63	<0.01

* The lowest measurable quantity of vitamin E was 7.0 nmol/g. Samples that measured below the level of detection were defined as 3.5 nmol/g.

The authors apologize for any inconvenience caused and state that the scientific conclusions are unaffected. The original publication has also been updated.
